# Very Low-Calorie Ketogenic Diet Modulates the Autonomic Nervous System Activity through Salivary Amylase in Obese Population Subjects

**DOI:** 10.3390/ijerph18168475

**Published:** 2021-08-11

**Authors:** Rita Polito, Anna Valenzano, Alessia Scarinci, Ines Villano, Marcellino Monda, Antonietta Messina, Giuseppe Cibelli, Chiara Porro, Ester La Torre, Daniela Pisanelli, Fiorenzo Moscatelli, Giovanni Messina, Vincenzo Monda

**Affiliations:** 1 Department of Advanced Medical and Surgical Sciences, Università degli Studi della Campania “Luigi Vanvitelli”, 80138 Naples, Italy; rita.polito@unicampania.it; 2 Department of Clinical and Experimental Medicine, University of Foggia, Viale Pinto, 71100 Foggia, Italy; anna.valenzano@unifg.it (A.V.); giuseppe.cibelli@unifg.it (G.C.); chiara.porro@unifg.it (C.P.); ester.latorre@unifg.it (E.L.T.); or daniela.pisanelli82@gmail.com (D.P.); fiorenzo400@gmail.com (F.M.); 3 Department of Education, Psychology, Communication, University of Bari, 70121 Bari, Italy; alessia.scarinci@uniba.it; 4 Section of Human Physiology and Unit of Dietetics and Sports Medicine, Department of Experimental Medicine, Università degli Studi della Campania “Luigi Vanvitelli”, 80138 Naples, Italy; ines.villano@unicampania.it (I.V.); marcellino.monda@unicampania.it (M.M.); antonietta.messina@unicampania.it (A.M.); vincenzo.monda@unicampania.it (V.M.)

**Keywords:** salivary amylase, heart rate variability (HRV), autonomic nervous system, obesity, very low-calorie ketogenic diet (VLCKD), visceral adipose tissue (VAT)

## Abstract

In obesity, to reduce visceral adipose tissue (VAT), caloric restriction is a valid strategy. Salivary amylase is an enzyme that cleaves large starch carbohydrates molecules and its production is modulated by the central nervous system. In addition, heart rate variability (HRV) is simply a measure of the variation in time between each heartbeat. This variation is controlled by the autonomic nervous system. In the light of this evidence, the aim of this study is to characterize the effect of a very low-calorie ketogenic diet (VLCKD) on the autonomic nervous system in obese patients. Twenty subjects affected by obesity were recruited before and after 8 weeks of VLCKD intervention to evaluate salivary amylase by the ELISA test and HRV analysis. These parameters significantly increased after dietary treatment, and positively correlate to each other. VLCKD exerts a positive effect on salivary amylase and HRV, ameliorating body composition and biochemical features. In brief, this dietary intervention improves the autonomic nervous system activity. This is the first study about the effects of VLCKD upon the autonomic nervous system, but further studies are needed to elucidate the mechanism undergone VLCKD effects.

## 1. Introduction

Saliva plays a fundamental role in human health, it protects the oral cavity, facilitates the digestion of food and also plays a role in the perception of taste. The perception of taste influences not only food choices, but also pre- and post-absorption physiological processes [1]. When we eat, we have what is called “cephalic phase responses” and these stimulate the body to efficiently metabolize the nutrients, playing an important role in food digestion and the prevention of dysglycemia and dyslipidemia. In addition, in saliva, there are various proteins involved in digestion activities such as lipases, peptidases and hydrolases. [2]. Among of these, α-amylase is the most abundant digestive enzyme in human saliva [2]. It cleaves large starch molecules into dextrins and maltodextrins in order to prepare carbohydrates for easier digestion and intestinal absorption of glucose [2]. The amylase is produced by the salivary glands and pancreas. Salivary and pancreatic amylases are structurally very similar but are encoded by different genes (AMY1 and AMY2, respectively) and have a different action on starches of various origins [1,2]. The physiological role of salivary amylase is still unclear, but it is known that there are advantages of generating higher levels of salivary amylase. Indeed, a higher production of amylase induces an early degradation of oral starch, ameliorating also the blood glycemic and insulin profile, as well as the disease states of metabolic syndrome, diabetes and obesity [1,2]. In addition, salivary amylase production is also modulated by the central nervous system. As is well known, the autonomic nervous system also has an action on the frequency of heartbeats, and the physiological variation between these heartbeats is used as an indirect measure of autonomic nervous system activity, which is known as heart rate variability (HRV) and is a parameter to evaluate sympathetic and parasympathetic modulation of the heart [3]. Many data in the literature report that there is a relationship between HRV and body mass index (BMI) in obese subjects [4,5]. In this scenario, it is well known that numerous obesity-related diseases can be counteracted with a proper diet and/or caloric restriction by reducing or slowing the onset of numerous inflammatory diseases and having innumerable beneficial effects by reducing oxidative stress [6]. For these reasons, nutritional interventions, such as caloric restriction diets, can be an effective therapeutic approach to promote weight loss in obese patients and increase salivary amylase and its beneficial effects [7]. The very low-calorie ketogenic diet (VLCKD) induces rapid weight loss and is also able to ameliorate hyperlipidemia and some cardiovascular risk factors; therefore, VLCKD has been shown to be a valid tool to counteract obesity in an average time frame of 3–6 months [8]. In light of this evidence, the present study aims to evaluate the effects of a commercial dietary ketosis program for weight loss on the autonomic nervous system through the assessment of salivary amylase levels and HRV analysis, to clarify the role of this enzyme also in anthropometric analysis and biochemical parameters involved in obesity prevention and a possible link between HRV and adiposity.

## 2. Materials and Methods

### 2.1. Subjects, Anthropometric and Biochemical Measurements

In total, 20 subjects affected by obesity (10 females, 10 males), aged between 20 and 60 years (mean 48 ± 10 years), were enrolled by the Laboratory of Physiology, Department of Clinical and Experimental Medicine, University of Foggia. The study was approved by local Ethics Committee 22 May 2018, n◦440/DS and conducted according to ethical principles of the Declaration of Helsinki. Written informed consent was obtained from all participants. The obese patients were subjected to a VLCKD intervention for 8 weeks, according to a commercial weight loss program (Lignaform, Therascience), consisting of <50 g/d carbohydrates from vegetables, 43% fat, 43% protein, 14% carbohydrates and totaling 700–900 kcal. The quantity of proteins ranged between 1 and 1.2 g per each kg of ideal body weight. Though the dietary intervention profile consisted of three different stages, for the purposes of this study, only the active ketogenic phases of the first stage were considered. Along this period, vitamins, minerals and Ω-3 fatty acids were provided in accordance with international recommendations. 

In our population, we evaluated body composition measuring height, weight, BMI and VAT by DEXA. Serum samples were collected after a 12-h overnight fasting period. Serum aliquots were stored at −80 °C. As previously reported, for all participants, glucose, total cholesterol, low-density lipoprotein (LDL), high-density lipoprotein (HDL), triglycerides, LDH, aspartate transaminase (AST), alanine transaminase (ALT), IL-10, tumor necrosis factor -a (TNF-a), adiponectin and Orexin-A were measured [9,10]. 

Interestingly, on salivary samples of the same population, we evaluated amylase levels. 

Salivary samples were collected between 9 and 11 a.m. twice, before the VLCKD intervention and after 8 weeks of VLCKD intervention, by means of cotton swabs (Salivette, Sarstedt, Rommelsdorf, Germany). Participants were asked to place the cotton swab into their mouth for at least 2 min while chewing and then insert it back into a special plastic tube. Samples were returned as soon as possible to the laboratory and stored at −20°C for salivary amylase assay. The absence of blood contamination was checked by a salivary blood contamination kit (Salimetrics LLC, State College, PA, USA). The saliva collecting tubes were centrifuged at 1500 g for 15 min at 4 °C. Then, 50 to 100 µL of saliva was used for duplicate analysis. All samples were tested in the same series to avoid any variations between tests. The amylase concentrations were measured by commercial kits (Sigma, Sydney, NSW, Australia, MAK009-1KT) according to the manufacturers’ instructions. A standard plate reader (Power Wave XS, Bio-Tech Instruments, Winooski, VT, USA) was used for salivary determination at 405 nm.

### 2.2. Study Protocol

As previously reported, all participants underwent a general medical examination [9]. To verify if the patients followed the diet correctly, every day ketone was measured by capillary blood (GD40 Delta test strips, TaiDoc Technology Co., New Taipei City, Taiwan). Nutritional ketosis was defined as a blood ketone (b-hydroxybutyrate) level >0.5 mmol/L. Fasting (12 h) blood samples were collected at 8:00 a.m. [9,10]. 

### 2.3. Heart Rate Variability Evaluation

Electrocardiogram (ECG) recordings were made before and after the VLCKD intervention. The experiments were conducted in the morning on an empty stomach. Obese patients were told not to consume beverages with caffeine for at least 12 h before the EGC and not to perform strenuous physical activity the previous evening. For assessment of resting HRV, subjects rested in a quiet room for at least 20 min. Electrodes were placed on the subject’s wrists in a supine three-lead position with the reference electrode placed on the right ankle. ECG activity was recorded for at least 5 min (sampling rate of 500 Hz) by the Biopac MP100 data acquisition system (Biopac System, Santa Barbara, CA, USA). HRV analysis was performed according to Triggiani et al. (2015) [11]. 

### 2.4. Statistical Analysis

Statistical analyses were performed using the StatView software 5.0.1.0 (SAS Institute, Cary, NC, USA). All data are presented as means ± SD. A *p*-value ≤ 0.05 was used for statistical significance. Salivary amylase, VAT, TNF-a, glycated hemoglobin, Orexin-A, IL-10 serum concentrations and HRV were correlated by Pearson’s or Spearman’s rho tests, according to data distribution. A *p*-value <0.05 was considered statistically significant. 

## 3. Results

### Salivary Amylase Evaluation and HRV Analyses in VLCKD Obese Patients

**Figure 1:**
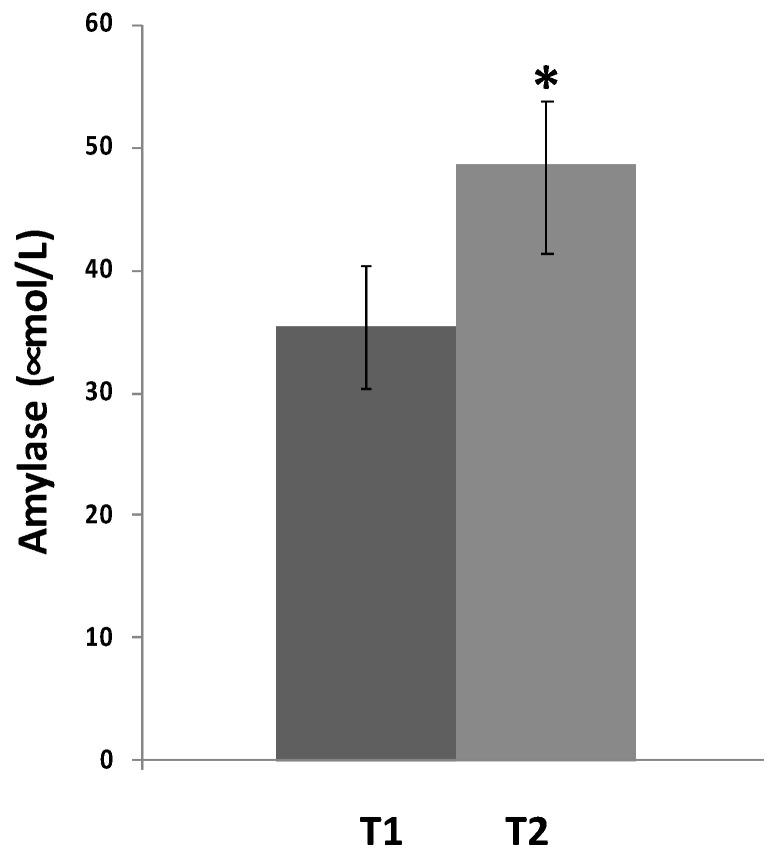
Salivary amylase levels before and after VLCKD in obese subjects. The Asterisk Indicates a Significant Difference (*p* < 0.05) compared to basal levels.

**Figure 2:**
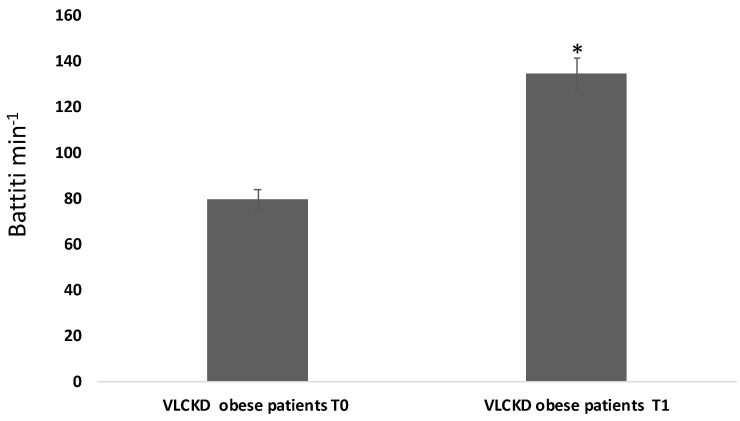
Heart rate variability analysis before and after VLCKD intervention. The Asterisk Indicates a Significant Difference (*p* < 0.05) compared to basal levels.

We found a statistical increase of amylase salivary levels in VLCKD obese patients before the diet intervention compared to same population of obese patients after the VLCKD intervention (Figure 1). In Figure 2, time and frequency domain HRV analyses are reported. As shown by the figure, HRV statistical increased in obese patients after VLCKD in the same condition of analysis.

**Figure 3:**
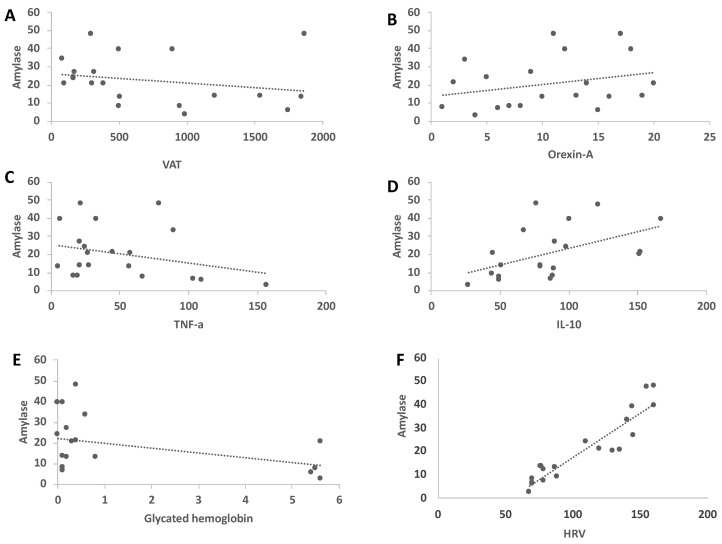
Salivary amylase correlated negatively with VAT, TNF-a and glycated hemoglobin (panels **A**,**C**,**E**) and positively correlated with Orexin-A,IL-10 and HRV (panels **B**,**D**,**F**).

Interestingly, we observed the correlations between salivary amylase, biochemical and anthropometric parameters in VLCKD obese subjects. In particular, salivary amylase negatively correlated with VAT, TNF-a and glycated hemoglobin, expressed as the D variation in Figure 3A,C,E. Furthermore, salivary amylase positively correlated with Orexin-A and IL-10 serum levels and HRV (Figure 3B,D,F).

## 4. Discussion

Obesity is a public health problem that affects all countries in the world, especially industrialized ones. It predisposes to an increased risk of onset of numerous cardiovascular and inflammatory diseases with a fatal course [12]. The prevalence of obesity is increasing worldwide at an alarming rate and approximately 30% of the world’s population is overweight or obese [13]. Therefore, a dietary intervention such as the ketogenic diet can have beneficial effects, in a short time, to counteract obesity. With this type of diet, there is not only a massive weight loss and better control of this process, but also a real physiological and biochemical change occurs in our body, improving not only the metabolic profile, but also cognitive functions. In this study, we analyzed the effects of VLCKD on salivary amylase levels in a population of obese subjects after 8 weeks of therapy. As we previously reported in obese subjects, after 8 weeks of the diet intervention, there was a strong modification in anthropometric parameters such as BMI, VAT and weight, and also in biochemical parameters such as glucose and lipid profiles, as well as in inflammatory status. In addition, many important proteins, such as adiponectin and Orexin-A, were strongly increased after 8 weeks of VLCKD, showing the important beneficial effects. The strength of this study is that, in a short period, VLCKD changed not only the anthropometric and metabolic profile, but also the autonomic nervous system activity of obese subjects. The biochemical composition of saliva is in some respects like that of plasma, but when comparing the proteomes of saliva and plasma, the distributions of salivary proteins are oriented toward metabolic and catabolic processes, indicating that saliva has an active physiological role in food digestion [14]. Regarding to salivary amylase, we still do not know all aspects of this enzyme, and we know that its regulatory secretory function in plasma remains a mystery [15].

Salivary amylase has a relatively short active contact time with starch. Once a food bolus is ingested and infiltrated with gastric juice, its catabolic activity is mostly interrupted by a low acidic pH. Some activity remains within the particles because of the barrier protection provided by the partially digested starch outside the particle [16], but most of the starch is digested by pancreatic amylase releasing by duodenum. However, studies have shown that significant hydrolysis of starch occurs within seconds in the oral cavity [17]. This change in consistency could affect starch digestion, sensory preferences and carbohydrate intake.

In our study, we found a modulation of salivary amylase in subjects affected by obesity before and after the VLCKD intervention. In the literature, not much is reported regarding the metabolic action of salivary amylase in obesity. Nevertheless, it is well known that serum amylase is closely correlated with obesity, type 1 and type 2 diabetes and metabolic syndrome [18]. Structurally, serum amylase is similar to salivary amylase, and data from the literature report that low serum amylase predisposes individuals to an increased risk of cardiovascular disease [19]. In addition, pancreatic amylase production is strongly influenced by insulin production, which we know plays a key role in diabetes and metabolic syndrome [20]. Schneeman reported that insulin resistance may influence pancreatic amylase production, and this would explain the lower levels of pancreatic amylase in diabetic subjects [21]. Regarding salivary amylase, we still do not know whether insulin can influence its production at the level of salivary acini in the mouth, but some studies indicate a causal action of insulin on salivary amylase production in salivary glands [22]. Therefore, there may be a direct functional link between insulin function and amylase production, thus creating a causal link between starch digestion, glucose homeostasis and metabolic syndrome. Furthermore, amylase is encoded by the AMY1 gene and recent studies have associated this gene with obesity in European and Asian populations [23,24]. In addition, the AMY1 gene is associated to a higher starch use, typical of obese conditions [25,26]. They found that increased salivary AMY1 copy number is positively associated with lower BMI and obesity risk, thus providing a genetic link between starch digestion efficiency and low BMI, due to the AMY1 gene. Furthermore, in children, the copy number of the salivary amylase gene (AMY1) is associated with obesity and inflammatory biomarkers in the children’s saliva sample. Indeed, AMY1 was significantly decreased and the obesity-related salivary biomarkers resistin, MCP-1, TNF-α, IL-6 and CRP were significantly increased in overweight/obese children compared with normal weight children [23,24,25]. Furthermore, Aldossari et al., 2019, according to our results, reported the relationship between AMY1 activity and anthropometric characteristics including weight, WC, HC and BMI in the control and overweight and obese groups, showing a significantly inverse relationship with weight, WC, HC and BMI in male and female adults in the overweight and obese group [25]. In addition, overweight and obese populations appear to be at risk for low AMY1 activity, which is related to their obesity. The AMY1 copy number variation is not the sole driver of amylase production in animals, since environmental factors such as stress level, circadian rhythms and diet [27] significantly contribute to quantitative variation among species and individuals. In addition, as reported by Mandel et al. it is possible that chronic high glucose level and insulin resistance, typical of obesity and induced by high starch intake, may elicit several hormonals, receptor and physiological changes that will indicate that individual differences in salivary amylase that may considerably contribute to overall nutritional status [26]. Considering this evidence, the VLCKD intervention is able, in a short time, to modify not only the anthropometric and metabolic profile of obese subjects, but also the salivary profile by modulating amylase production. This enzyme is very important, in fact, as reported by Peyrot des Gachons et al., 2019, salivary amylase influences the oral perception of carbohydrate taste, metabolic signaling in the pre-absorption phase and plasma glucose responses to ingested starch. Interestingly, in humans during evolution, with agriculture, the consumption of carbohydrates and thus starch has greatly increased, and because of this, the salivary amylase gene has greatly expanded as a copy number variant. However, today, we tend to eat the same amounts of carbohydrates regardless of whether we produce high or low levels of salivary amylase. Therefore, those who produce low levels of salivary amylase and eat high amounts of carbohydrates are at risk of developing metabolic syndrome [27]. In addition, Nakajima et al., 2020, reported that fatty acids may be used predominantly as an energy source in individuals with low serum amylase; it is unclear whether a lipid-rich diet is suitable for individuals with low serum amylase [28]. Taken together, there may be a relationship between serum amylase and the specific type of macronutrient burning used to produce energy. Although increases in serum amylase occur due to leakage from the salivary glands and pancreas, the clinical relevance of this remains unknown. Circulating amylases may be only a marker of leakage or damage, although several investigators have suggested a feedback system between serum amylase and insulin action [28]. Insulin resistance may reduce amylase production [29], probably for the purpose of reducing the absorption of glucose digested from starch. Conversely, high serum amylase levels may reduce insulin secretion in the pancreas [30]. However, it is not known whether this plausible feedback system is also applicable in the salivary gland [30,31]. Furthermore, in our study, we found the negative correlation between salivary amylase and VAT, and glycated hemoglobin and TNF-a, confirming the important involvement of salivary amylase in the occurrence of many metabolic and inflammatory processes such as obesity and metabolic syndrome, also demonstrating the short-term beneficial effects of the VLCKD intervention not only in the treatment of obesity, but also in the establishment of obesity-related diseases such as cardiovascular disease, metabolic syndrome or insulin resistance [32,33,34]. Interestingly, salivary amylase is modulated by the central nervous system; therefore, in our study, the increase in HRV after the VLCKD and its positive correlation with salivary amylase and serum Orexin-A levels, indicates a modulation of the autonomic nervous system by VLCKD intervention. The positive correlation between salivary amylase and HRV that we found is also corroborated by many data from the literature that report how these two factors are expressions of the central nervous system and how they are modulated by stress factors such as a dietary diet or physical activity [35,36,37]. For this reason, the expression of salivary amylase and HRV are not only modulated by the central nervous system in response to dietary stress, but also by a reduced BMI, which is inversely related to the production of Orexin-A. It is well known that all these factors are also strongly influenced by body composition, and with a negative feedback mechanism, they influence each other by acting on central nervous system activity [9]. Indeed, as reported by Triggiani et al., 2015, there is an association between increased body fat and hypoactivity of the sympathetic and parasympathetic components of the autonomic nervous system on the heart, although the reduction of sympathetic activity in obese subjects is controversial [3]. Furthermore, it has been hypothesized that the central distribution of body fat may be more important than overall adiposity because of its metabolic activity [38,39,40]. Furthermore, as reported previously by Young et al., HRV is reduced in undernourished people. Subsequently, these findings were also confirmed in lean young people [32,41]. The relationship between HRV and BMI was studied by Wu et al., who, in contrast, found a reduction in HRV in obese subjects, compared with normal weight subjects. Recently, a study found a reduction in HRV frequency parameters in obese young adults [42]. Furthermore, although insulin and Orexin-A act on the brain to modulate food intake, energy expenditure and energy storage in adipose tissue, other signals may be involved. Neural and metabolic signals have been proposed as a mechanism for the brain to sense the energy status of the body [43]. In this respect, glucose has been proposed as playing a significant role because neurons, whose activity is modified by changes in glucose, and which have been described in areas associated with neuroendocrine and autonomic control systems [44].

## 5. Conclusions

The VLCKD has a positive effect on salivary amylase production and HRV. In addition, the VLCKD improves the anthropometric and metabolic profile of these obese subjects. Interestingly, over a short period, this dietary intervention also has an action on autonomic nervous system activity. The increase of the autonomic nervous system through salivary amylase production and HRV is important for the positive outcome of the diet intervention. The modulation of the autonomic nervous system is by a modification of body composition and by a modification in hormonal factors, such as Orexin-A, which leads to increased salivary amylase production and HRV. For these reasons, these two factors could be used as rapid and non-invasive biomarkers to determine the activation of the autonomic nervous system and in the dietary regimen, and they can also be applied to evaluate the progress of dietary therapy. Finally, to the best of our knowledge, this is the first study of the effects of the VLCKD on the autonomic nervous system, supporting the utility of such a therapeutic intervention in promoting the reduction of individual disease burden.

## Data Availability

Data is contained within the article. Authors can use this data for research purposes only by citing our research article.
